# S100B Inhibition Attenuates Intestinal Damage and Diarrhea Severity During *Clostridioides difficile* Infection by Modulating Inflammatory Response

**DOI:** 10.3389/fcimb.2021.739874

**Published:** 2021-09-10

**Authors:** Deiziane V. S. Costa, Vivaldo Moura-Neto, David T. Bolick, Richard L. Guerrant, Jibraan A. Fawad, Jae H. Shin, Pedro H. Q. S. Medeiros, Solanka E. Ledwaba, Glynis L. Kolling, Conceição S. Martins, Venkat Venkataraman, Cirle A. Warren, Gerly A. C. Brito

**Affiliations:** ^1^Department of Physiology and Pharmacology, Faculty of Medicine, Federal University of Ceará, Fortaleza, Brazil; ^2^Division of Infectious Diseases and International Health, University of Virginia, Charlottesville, VA, United States; ^3^Department of Morphology, Faculty of Medicine, Federal University of Ceará, Fortaleza, Brazil; ^4^Paulo Niemeyer Brain Institute, Federal University of Rio de Janeiro, UFRJ, Rio de Janeiro, Brazil; ^5^Department of Microbiology, Faculty of Medicine, Federal University of Ceará, Fortaleza, Brazil; ^6^Department of Biochemistry and Microbiology, Faculty of Science, Engineering and Agriculture, University of Venda, Thohoyandou, South Africa; ^7^Department of Cell Biology and Neuroscience, Rowan University School of Osteopathic Medicine, Stratford, NJ, United States; ^8^Department of Rehabilitation Medicine, Rowan University School of Osteopathic Medicine, Stratford, NJ, United States

**Keywords:** *Clostridioides difficile*, S100B, inflammation, diarrhea, enteric glia

## Abstract

The involvement of the enteric nervous system, which is a source of S100B, in *Clostridioides difficile* (*C. difficile*) infection (CDI) is poorly understood although intestinal motility dysfunctions are known to occur following infection. Here, we investigated the role of S100B in CDI and examined the S100B signaling pathways activated in *C. difficile* toxin A (TcdA)- and B (TcdB)-induced enteric glial cell (EGC) inflammatory response. The expression of S100B was measured in colon tissues and fecal samples of patients with and without CDI, as well as in colon tissues from *C. difficile*-infected mice. To investigate the role of S100B signaling in *IL-6* expression induced by TcdA and TcdB, rat EGCs were used. Increased S100B was found in colonic biopsies from patients with CDI and colon tissues from *C. difficile-*infected mice. Patients with CDI-promoted diarrhea exhibited higher levels of fecal S100B compared to non-CDI cases. Inhibition of S100B by pentamidine reduced the synthesis of IL-1β, IL-18, IL-6, GMCSF, TNF-α, IL-17, IL-23, and IL-2 and downregulated a variety of NFκB-related genes, increased the transcription (SOCS2 and Bcl-2) of protective mediators, reduced neutrophil recruitment, and ameliorated intestinal damage and diarrhea severity in mice. In EGCs, TcdA and TcdB upregulated *S100B-*mediated *IL-6* expression *via* activation of RAGE/PI3K/NFκB. Thus, CDI appears to upregulate colonic S100B signaling in EGCs, which in turn augment inflammatory response. Inhibition of S100B activity attenuates the intestinal injury and diarrhea caused by *C. difficile* toxins. Our findings provide new insight into the role of S100B in CDI pathogenesis and opens novel avenues for therapeutic interventions.

## Introduction

*Clostridioides (*formerly, *Clostridium) difficile (C. difficile)* is an obligate anaerobic, spore-forming gram-positive bacillus that is able to colonize, germinate, and proliferate in the human gut after antibiotic use (Martin et al., 2016). The incidence of *C. difficile* infection (CDI) worldwide has increased. In European hospitals, seven CDI cases occur for every 10,000 overnight patients (Davies et al., 2014; Peery et al., 2019). CDI is the 10th leading cause of readmissions within 30 days for gastrointestinal disease and the fifth leading cause of death from nonmalignant gastrointestinal disease in the USA, costing approximately $US 4 billion per year (Peery et al., 2019). The clinical disease ranges from mild diarrhea to toxic megacolon, colonic perforation, or death (Solomon, 2013; Walker et al., 2013).

Germination of *C. difficile* spores resulting in toxin production within the gut lumen leads to development of CDI. *C. difficile* toxin A (TcdA) and B (TcdB) are the main toxins produced by *C. difficile*, and they are able to disrupt the colonic epithelial barrier, activate immune cells, and stimulate the release of proinflammatory cytokines and chemokines (Solomon, 2013).

It has been shown that TcdA and TcdB stimulate the release of IL-1β, IL-6, TNF-α, IL-8, IL-12, IL-18, IL-23, C–C motif chemokine 4 (also known as macrophage inflammatory protein 1β), C–X–C motif chemokine 2 (also known as macrophage inflammatory protein 2α), and leptin macrophage migration inhibitory factor (MIF) from epithelial cells, immune cells, or enteric neurons (Ng et al., 2010; Buonomo et al., 2013; Yu et al., 2017; Jose et al., 2018). This inflammatory response is a major determinant of disease severity (Kelly and Kyne, 2011; Yu et al., 2017) and has been shown to correlate with persistent diarrhea and poor clinical outcome (El Feghaly et al., 2013; Walker et al., 2013).

There is growing evidence suggesting that dysregulation of enteric glial cells (EGCs) can contribute to intestinal dysmotility (which can lead to diarrhea) and immune responses during bowel inflammatory diseases (Grubišić et al., 2018). Intestinal dysmotility or functional diarrhea has been identified in patients after CDI (Gutiérrez et al., 2015). Glial fibrillary acidic protein (GFAP), an enteric glial marker, has been noted to be increased in colonic tissue from patients with CDI (von Boyen et al., 2011). Other studies have demonstrated that TcdB induces apoptosis (Fettucciari et al., 2017; Macchioni et al., 2017) and senescence in EGCs (Fettucciari et al., 2018).

EGCs can release a variety of mediators, such as IL-6, IL-1β, nitric oxide (NO), and S100 calcium-binding protein B (S100B) (Cirillo et al., 2011; Macchioni et al., 2017). Among these, S100B has been broadly investigated because it works as a proinflammatory cytokine during inflammation, activating a cascade of signaling pathways, such as the receptor for advanced glycation end products (RAGE)/phosphoinositide 3-kinase (PI3K)/nuclear factor-kappa B (NFκB) and RAGE/signal transducer and activator of transcription 3 (STAT3) pathways, as previously reported in other cell types (Villarreal et al., 2011; Zhang et al., 2011). However, the role of S100B during CDI and whether TcdA and TcdB promote S100B secretion in EGCs remain unclear.

In this study, we determined whether intestinal S100B is upregulated in *C. difficile-*infected mice and human and investigated whether the S100B signaling pathway may be involved in intestinal damage and diarrhea during CDI, as well as in EGC proinflammatory response.

## Material and Methods

### Clinical Samples

After Institutional Review Board (IRB) protocol approval (Protocol number: 21079), deidentified intestinal surgical resection specimens in paraffin blocks were obtained from the biorepository and tissue research facility of the University of Virginia (UVA, USA).

Fecal samples from patients with diarrhea caused by CDI and non-CDI were obtained from the clinical microbiology laboratory of University of Virginia after Institutional Review Board (IRB) protocol approval (Protocol number: 20813).

### Mice

A total of 48 male C57BL/6 mice (Jackson Laboratory, Farmington, USA, 8 weeks of age) were housed in temperature-controlled rooms under 12-h light–dark cycles. The animals received water and food *ad libitum*. All surgical procedures and treatments performed with C57BL/6 mice were conducted in accordance with the Guidelines for Institutional and Animal Care and Use of the University of Virginia, Charlottesville, USA. The protocol has been approved by the committee on the Ethics of Animal Experiments of the University of Virginia (Protocol number: 4096).

### *C. difficile* Infection Model

Our murine CDI model was established as previously described (Chen X. et al., 2008; Moore et al., 2015; Shin et al., 2017; Broecker et al., 2019; Schmidt et al., 2020). This model is broadly used due to the ability to mimic clinical symptoms, such as severe diarrhea, presented by humans with CDI (Chen X. et al., 2008; Moore et al., 2015; Shin et al., 2017; Broecker et al., 2019; Schmidt et al., 2020). C57BL/6 mice (n = 5 for each group) received an antibiotic treatment in the drinking water for 3 days. The antibiotic cocktail contained gentamicin (0.035 mg per ml), colistin (850 U per ml), metronidazole (0.215 mg per ml), and vancomycin (0.045 mg per ml). After 1 day off antibiotics, an intraperitoneal injection of clindamycin (32 mg per kg) was given 1 day before *C. difficile* challenge. Then, 10^5^ CFU (in 100 μl of chopped meat broth, a pre-reduced medium) of the vegetative *C. difficile* strain VPI10463 (ATCC 43255, *tcdA+tcdB+cdtB-*) was administered by oral gavage. Control mice received chopped meat broth (100 μl). Mouse weights and the development of disease symptoms were monitored daily. Animals that became moribund or lost >20% of their body weight were euthanized.

For the S100B inhibitor treatment, mice were injected intraperitoneally daily once a day, starting 1 h prior *C. difficile* inoculation, with pentamidine (4, 10, or 40 mg/kg, Sigma-Aldrich, P0547) or saline solution.

### Rat Enteric Glial Cell Culture and Treatment

The immortalized rat enteric glial cell (EGC) line PK060399egfr (ATCC CRL-2690, VA, USA) was cultured in Dulbecco’s modified Eagle’s medium (DMEM, Gibco) and supplemented with 10% fetal bovine serum, 1% antibiotics (100 μg/ml penicillin and 100 μg/ml streptomycin, Gibco), and 1 mM sodium pyruvate (Gibco) at 37°C in a humidified incubator under 5% CO_2_ for no more than 16 passages. For all experiments, EGCs were released using 0.05% trypsin-EDTA for 5 min.

Cells were incubated with TcdA (10, 50, or 100 ng/ml) or TcdB (0.1, 1, or 10 ng/ml) for 0.5, 2, 6, 12, 18, 24, and 48 h to establish the best time point to study each parameter. For some mechanistic studies, cells were incubated with 30 μM FPSZM1 (TOCRIS, 6237), a high-affinity antagonist of RAGE, and a PI3K inhibitor (1 and 10 μM LY294002, TOCRIS, 1130) 1 h before incubation with TcdA or TcdB. All drug concentrations used were based on MTT assay results, as shown in Additional file (**Additional File: Figure S1**).

Purified TcdA and TcdB, produced by *C. difficile* strain VPI10463, were obtained from TechLab (VA, USA).

The EGC lineage (EGC/PK060399) has been shown to exhibit similar morphology and functional properties to primary enteric glial cells (Ruhl et al., 2001). This EGC lineage expresses GFAP and S100B, which are enteric glial factors, and has been applied in a variety of mechanistic studies (Bauman et al., 2017; Fettucciari et al., 2017; Macchioni et al., 2017; Chen et al., 2018; Fettucciari et al., 2018; Kimono et al., 2019).

### Immunohistochemistry

Sections (4 µm thick) were prepared from paraffin-embedded mouse and human colonic tissues. After deparaffinization, antigens were recovered by incubating the slides in citrate buffer (pH 6.0) for 20 min at 95°C. Endogenous peroxidase was blocked with 3% H_2_O_2_ for 10 min to reduce non-specific binding. The sections were then incubated with an S100B antibody (NBP2-54426, Novus Biologicals, 1:1,000) overnight. The sections were then incubated for 30 min with polymer (K4061, Dako). The antibody-binding sites were visualized by incubating the samples with diaminobenzidine–H_2_O_2_ (DAB, Dako) solution. Sections incubated with antibody diluent without a primary antibody were used as negative controls. Antibody specificity was evaluated using positive controls for S100B in the mouse cerebellum (data not shown). The immunostaining for S100B in human samples was performed at the UVA Biorepository and Tissue Research Facility.

The amounts of DAB products after immunostaining were estimated from at least 6–8 and 15–20 digital images from different areas of each section (from four specimens per group) for mouse and human samples, respectively, at ×400 magnification using Adobe Photoshop software. The percentage of immunopositive area was calculated by dividing the number of DAB-positive pixels (immunostaining-positive pixels) by the number of pixels per total tissue image and multiplying the result by 100, as previously described (Costa et al., 2019).

### S100B Western Blot Analysis

Proteins from colon tissue from CDI-infected and control mice at days 1 and 3 p.i. were extracted by lysing the tissues using RIPA lysis buffer (supplemented with complete EDTA-free protease inhibitor cocktail and PhosSTOP, Sigma-Aldrich), followed by a step of centrifugation (17 min, 4°C, 13,000 rpm) and collection of the supernatant. Protein concentrations were determined through the bicinchoninic acid assay according to the manufacturer’s protocol (Thermo Fisher Scientific). Reduced 40-µg protein samples (prepared with a sample reducing agent—Invitrogen—and a protein loading buffer—LI-COR) were denatured at 99°C for 5 min, separated on NuPAGE 4%–12% BIS-Tris gel (Invitrogen), and transferred to nitrocellulose membranes (Life Technologies) for 2 h. The membranes were then immersed in iBind fluorescent detection solution (Life technologies) and placed in an iBind automated Western device (Life Technologies) overnight at 4°C for blocking and incubating with primary antibodies (mouse anti-α-tubulin, 1:2000, Sigma-Aldrich; rabbit anti-S100B, 1:500, Novus Biologicals, NBP2-54426) and secondary antibodies (Cy3-conjugated AffiniPure donkey anti-rabbit, 711-165-152, 1:1000, Jackson ImmunoResearch, and Cy5-conjugated AffiniPure donkey anti-mouse, 715-175-150, 1:1000, Jackson ImmunoResearch). Then, the membranes were immersed in ultrapure water and fluorescent signal was detected using the Typhoon system (GE Healthcare). Densitometric quantification of bands was performed using ImageJ software (NIH, Bethesda, MD, USA).

### *C. difficile* Shedding in Stools

For stool shedding of *C. difficile*, DNA was extracted from stools using the QIAamp DNA Stool Mini Kit (Qiagen) according to the manufacturer’s instructions. The *tcdB* (*C. difficile* toxin B) gene was used as a specific target for detecting *C. difficile* in stools after CDI. Primer sequences included *tcdB* 5′-AATGCATTTTTGATAAACACATTG-3′ (forward) and 5′-AAGTTTCTAACATCATTTCCAC-3′ (reverse). Real-time PCR was performed using Bio-Rad CFX under the following conditions: 95°C for 3 min, followed by 40 cycles of 15 s at 95°C, 60 s at 55°C, and lastly 20 s at 72°C.

### Diarrhea Analysis

Diarrhea, an important CDI outcome, was assessed at days 2 and 3 p.i. Diarrhea scores were based on the following 0 to 3: 0—well-formed pellets; 1—stick stools adhering in microtube wall or color change (yellow); 2—pasty stools with or without mucus; and 3—watery stools, as previously described (Warren et al., 2013) with some modifications.

### Measurement of Microscopic Damage

Mouse cecum and colon tissues from day 3 p.i. were fixed in 10% neutral buffered formalin for 20 h, dehydrated, and embedded in paraffin. Cecal and colonic sections (5 µm) were then stained with hematoxylin and eosin staining (H&E) and examined using light microscopy. Histopathological scores were performed by a blinded investigator, using a previously described method (Ledwaba et al., 2020) with some modifications. Histopathological scores were determined by quantifying the intensity of epithelial tissue damage (0–3, 0—no damage, 1—mild, 2—moderate, 3—extensive), edema in mucosa/submucosa layer (0–3), and cell infiltration (0–3). The total histological damage score was measured by the sum of the three parameters evaluated.

### Cytokine Analysis

Protein lysates were extracted from the cecum contents, cecum, and colon tissues using radioimmunoprecipitation assay (RIPA) buffer (20 mM Tris, 150 mM NaCl, 1% Nonidet P-40, 0.5% sodium deoxycholate, 1 mM EDTA, 0.1% SDS, adjusted to pH 7.5) containing protease inhibitor cocktail (Sigma-Aldrich) and phosphatase inhibitors (Sigma-Aldrich). Lysates were centrifuged at 13,000 rpm for 15 min, and the supernatant was used to perform the protein assay using the bicinchoninic acid assay (Thermo Fisher Scientific). Inflammatory biomarkers (MPO, IL-23, IL-22, IL-17, GMCSF, and IL-33) were measured using a commercial ELISA kit (R&D Systems) according to the manufacturer’s instructions. The absorbance (450 nm) was determined using an Epoch plate reader (BioTek). Interleukin-6 (IL-6), IL-1β, TNF-α, IL-18, and IL-2 were measured using a ProcartaPlex multiplex immunoassay (Invitrogen) by Luminex (Bio-Rad). Levels of these cytokines were measured as picograms per milligram of protein.

### TaqMan Real-Time Polymerase Chain Reaction (qPCR)

The isolation of total RNA from colon tissues of CDI-infected and control mice was performed by using a Qiagen RNeasy Mini Kit and QIAcube. cDNA was synthetized from 1 µg of total RNA, quantified by Qubit 3 Fluorometer 3000 (Invitrogen), and purified by deoxyribonuclease I (Invitrogen) treatment, with the iScript cDNA (Bio-Rad) as described by the manufacturer’s instructions. qPCR was performed with 50 ng of cDNA in each well and SensiFAST Probe No-ROX Mix (Bioline) using a CFX Connect system (Bio-Rad) with the following conditions: 95°C for 2 min, 40 cycles of 95°C for 10 s, and 60°C for 50 s. A predesigned TaqMan array mouse immune fast 96-well plate (Applied Biosystems) was used to assess the expression of all the genes shown in **Figures 4A, B**. Glyceraldehyde-3-phosphate dehydrogenase (GAPDH) was used as a reference gene. All fold changes were determined using the ΔΔC_t_ method (Livak and Schmittgen, 2001).

### MTT Assay

EGC lines (5×10^3^ cells/well) were seeded in 96-well plates and treated with TcdA or TcdB for different incubation periods. Then, the cells were incubated with thiazolyl blue tetrazolium bromide (MTT, 0.5 mg/ml reconstituted in supplemented DMEM, Sigma-Aldrich, M2128) for 2 h at 37°C in a humidified incubator under 5% CO_2_. After removal of the MTT solution, 150 µl of dimethylsulfoxide was added to each well. The plates were then shaken for 2 min at room temperature, and the absorbance of the reaction at 570 nm was measured using an ELISA reader.

### Cell Morphology Analysis

EGC lines (5×10^3^ cells/well) were seeded in 96-well plates and treated with TcdA or TcdB for different incubation periods. The percentage of rounded cells was measured among 100 cells from the center of the well, and rounded versus non-rounded cells were discriminated using contrast microscopy.

### Measurement of S100B Protein

Fecal samples were homogenized with RIPA buffer (1:2), and the proteins were obtained by centrifuging (10,000 rpm, 10 min, 4°C). Levels of S100B were measured with a DuoSet S100B kit (R&D Systems) by ELISA according to the manufacturer’s protocol. The absorbance (450 nm) was determined using an Epoch plate reader (BioTek). The range of S100B detection was 46.9–3,000 pg per mg of protein.

EGCs line (6×10^5^ cells/well) were seeded in six-well plates and treated with TcdA or TcdB for different times. EGC supernatants were collected (stored at -80°C until use) and centrifuged (10,000 rpm, 10 min, 4°C), and secreted S100B was measured with a DuoSet S100B kit (R&D Systems) by ELISA according to the manufacturer’s protocol. The absorbance (450 nm) was determined using an Epoch plate reader (BioTek). The range of S100B detection was 46.9–3,000 pg/ml.

### Quantitative Real-Time PCR

EGC lines (6×10^5^ cells/well) were seeded in six-well plates and treated with TcdA or TcdB and pharmacologic modulators. After incubation, total RNA was extracted with an RNeasy Plus Mini Kit (Qiagen, Hilden, Germany) using QIAcube (Qiagen). RNA was quantified with a Qubit 3.0 fluorometer (Life Technologies) using a Qubit RNA BR Assay Kit (Invitrogen, Q10211). After DNA contamination was removed by RNA treatment with DNase I (Invitrogen, 18068-015), a total of 600 ng of RNA was then reverse transcribed using an iScript cDNA Synthesis Kit (Bio-Rad, 1708891) according to the manufacturer’s protocol. qPCR amplification of *S100B*, *IL-6*, *RAGE*, and *glyceraldehyde 3-phosphate dehydrogenase* (*GAPDH*) in cell samples was performed in a CFX Connect system (Bio-Rad) with the following conditions: 95°C for 30 s, 40 cycles of 95°C for 5 s and 60°C for 30 s, and melt curve analysis from 65 to 95°C in 0.5°C increments for 2 s each. All PCRs were performed with iTaq Universal SYBR Green Supermix (Bio-Rad, 172-5124). The primer sets are listed in **Additional File: Table S1**.

### Immunofluorescence

EGC lines (4×10^4^ cells/well) plated on eight-chamber glass tissue culture slides in a polystyrene vessel and treated with TcdA or TcdB for 18 h were fixed in 4% PFA solution (Alfa Aesar) in PBS for 30 min at room temperature and permeabilized with 0.5% Triton X-100 (Sigma-Aldrich) and 3% bovine serum albumin (BSA, Sigma) in PBS for 10 min at 4°C. After blocking with 5% normal donkey serum (Jackson ImmunoResearch, 017-000-121) in PBS for 30 min at room temperature, the cells were incubated with anti-NFκBp65 (Santa Cruz Biotechnology, sc-372) overnight at 4°C. After three washes with washing buffer (0.01% Tween 20 in PBS), the cells were incubated for 1 h with Cy3-conjugated AffiniPure donkey anti-rabbit (Jackson ImmunoResearch, 711-165-152) antibody, washed with PBS, and mounted with ProLong Gold antifade reagent containing DAPI (Thermo Scientific, P36931). The samples were visualized by fluorescence microscopy (Zeiss). For each experimental condition, 100 cells were counted, and the percentage of cells (%) with positive nuclear staining for NFκBp65 was determined.

### Measurement of Nuclear NFκBp65 and Phosphorylated NFκBp65

EGC lines (6×10^5^ cells/well) were seeded in six-well plates and treated with TcdA or TcdB and pharmacologic modulators. After incubation, the supernatant was removed, and the nuclear extract was obtained by using a Nuclear Extract Kit (Thermo scientific) according to the manufacturer’s protocol. Protein concentrations were determined through the bicinchoninic acid assay according to the manufacturer’s protocol (Thermo Fisher Scientific). Reduced 15-µg protein samples (previously prepared with a sample reducing agent—Invitrogen NP0009—and a protein loading buffer—LI-COR 928-40004) were denatured at 99°C for 5 min, separated on NuPAGE 10% BIS-Tris gel (Invitrogen, NP0322BOX), and transferred to nitrocellulose membranes (Life Technologies, LC2000 or LC2006) for 2 h. After blocking with 5% non-fat dry milk at 4°C for 1 h, the membranes were incubated overnight with primary antibodies (mouse PCNA, 1:200, Cell Signaling; rabbit anti-NFκBp65, sc-372, 1:500, Santa Cruz Biotechnology; mouse laminin B1, sc-365962, 1:100, Santa Cruz Biotechnology; rabbit anti-phosphorylated NFκBp65, PA5-37718, 1:500, Thermo Scientific) and secondary antibodies (Cy3-conjugated AffiniPure donkey anti-rabbit, 711-165-152, 1:1000, Jackson ImmunoResearch; Cy5-conjugated AffiniPure donkey anti-mouse, 715-175-150, 1:1000, Jackson ImmunoResearch) for 1 h and 30 min. The membranes were washed in Tris-buffered saline (TBS) containing 0.05% Tween 20 (TSB-T), and the fluorescent signal was detected using the Typhoon system (GE Healthcare). Densitometric quantification of bands was performed using ImageJ software (NIH, Bethesda, MD, USA).

### Statistical Analysis

Analyses were performed using GraphPad software 9.0 (San Diego, CA, USA). The data are presented as the mean ± standard error of the mean (SEM). Student’s t test or one- or two-way analysis of variance (ANOVA) followed by the Bonferroni test was used to compare means. *p* < 0.05 was considered to indicate significance.

## Results

### S100B Expression Is Increased in Fecal Samples and Colonic Tissues From *C. difficile*-Infected Patients and Mice

**Figure 1:**
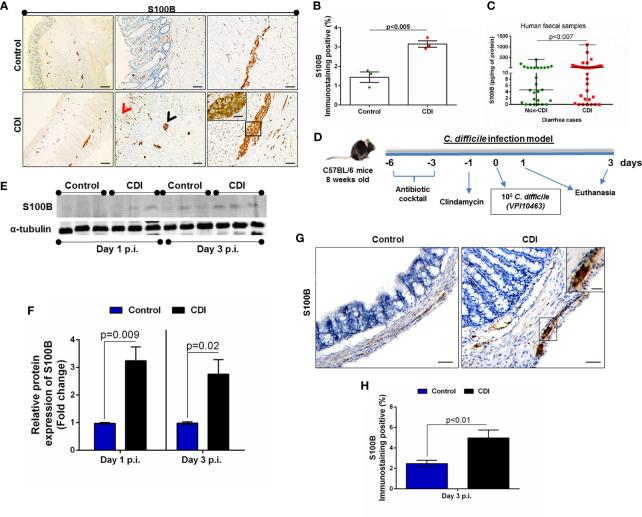
*C. difficile* infection increases S100B in fecal samples and in colon from humans and mice. **(A)** Representative immunohistochemical images of S100Bexpression in human colonic biopsies with active *C. difficile* infection (CDI) and healthy subjects (Control). Increased S100B expression (arrowhead or rectangle) was found in colonic mucosal (center panel, red arrowhead), submucosal (center panel, black arrowhead), and myenteric plexus (right panel, black rectangle). Scale bars, 200 (left panels), 100 (center panels), and 50 (right panels) μm. Left panels are showing colon tissues from control and CDI patients in a low magnification. **(B)** Quantification of percentage (mean ± s.e.m.) of S100B-immunopositive area in colons from human control and *C. difficile-*infected subjects in 15–20 microscope fields per sample (n = 4 subjects per group). Unpaired two-tailed Student t test. **(C)** S100B levels in fecal samples of patients with diarrhea caused by CDI (n = 53) and non-CDI (n = 27) evaluated by ELISA. Data are median ± s.d. Two-tailed non-parametric Mann–Whitney U-test. **(D)** Schematic diagram of CDI experimental model in mice. **(E)** Representative Western blot (WB) bands of S100B and α-tubulin in colonic tissues from mice infected with *C. difficile* (CDI group) and non-infected (control group) at days 1 and 3 postinfection (p.i.). **(F)** WB analysis of S100B (mean ± s.e.m.) in colonic tissues from CDI and control group (n = 3 mice per group). α-Tubulin was used to normalize the levels of S100B. Unpaired two-tailed Student’s t test. **(G)** Representative immunohistochemical images of S100B immunostaining in colonic tissues of mice with CDI and control (non-infected mice). **(H)** Quantification of percentage (mean ± s.e.m.) of S100B-immunopositive area in colon from mice with CDI and control (non-infected mice) (n = 5 mice per group). Unpaired two-tailed Student’s t test.

Increased S100B immunostaining was observed in colon mucosal, submucosal, and myenteric plexus tissues from patients with CDI (**Figure 1A**), and these results were verified by increased percentages of S100B-immunopositive cells in patients with CDI compared to control subjects (p < 0.0001, **Figure 1B**). In addition, we found increased S100B in fecal supernatants from patients with diarrhea associated with CDI compared to diarrhea not associated with CDI (**Figure 1C**). When age and gender were analyzed, we found that age 40–59 years and female gender had the statistically significant increases in levels of S100B among these patients with diarrhea associated with CDI compared to diarrhea not associated with CDI (**Additional File: Figures S2A, B**). In addition, no statistical difference was found between females and males with diarrhea associated with CDI (**Additional File: Figure S2B**).

Given that CDI murine models (**Figure 1D**) are important for investigating the pathogenesis of *C. difficile*-induced disease, we analyzed colonic tissues for S100B protein expression in this experimental model on days 1 and 3 postinfection (p.i.). We found that CDI increased the levels of colonic S100B on day 1 (p = 0.009) and day 3 (p = 0.02) p.i. compared to uninfected mice (**Figures 1E, F**).

Like patients with CDI, we found increased S100B in the colonic mucosa, submucosa, and myenteric plexus in mice with CDI on day 3 p.i. (**Figure 1G**). Immunostaining showed that CDI markedly enhanced the S100B protein expression in the colon of mice with CDI compared to the control group (p < 0.0001, **Figure 1H**).

These data demonstrated the association of elevated S100B expression with CDI.

### S100B Inhibition Decreases Disease Severity and Intestinal Epithelial Injury During CDI in Mice

**Figure 2:**
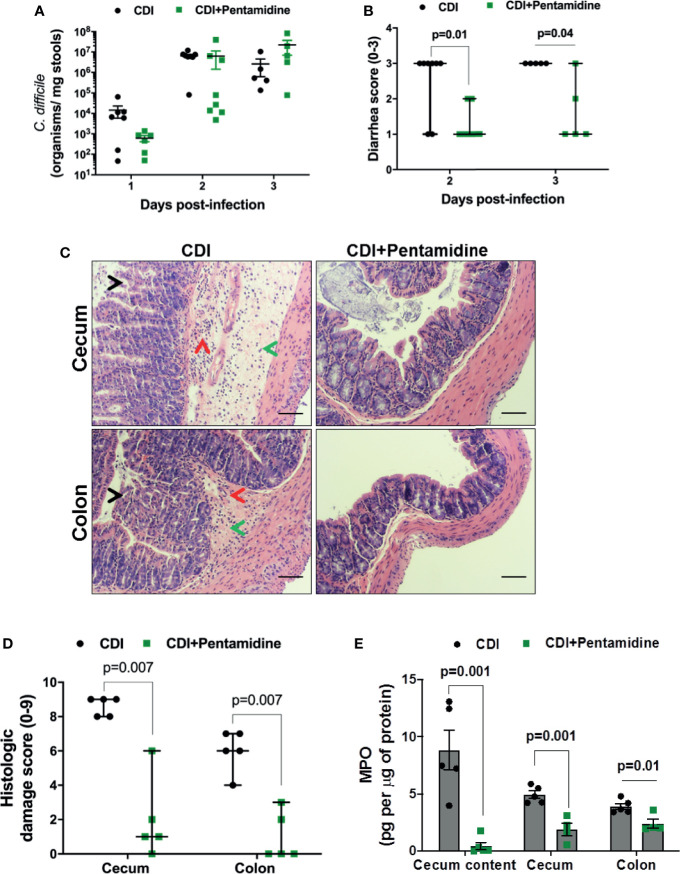
S100B inhibition decreases diarrhea severity, intestinal damage, and neutrophil recruitment during CDI in mice. Mice were infected with 10^5^ vegetative *C. difficile* (VPI10463 strain) and treated with a S100B inhibitor, pentamidine (40 mg/kg, i.p., once daily for 3 days, CDI+pentamidine group) or non-treated (CDI group). **(A)** Quantification of *C. difficile* shedding (mean ± s.e.m.) in stools by amplifying the *tcdB* gene by qPCR. Unpaired two-tailed *Student’s t* test. **(B)** Diarrhea score (median ± s.d) of CDI and CDI+pentamidine mice. Two-tailed non-parametric Mann–Whitney U-test. **(C)** Representative H&E stains of cecal and colonic tissues collected from CDI and CDI+pentamidine mice at day 3 postinfection (p.i.). CDI promotes damage of colonic and cecal epithelium (black arrow), edema (green arrow), and inflammatory cell infiltration (red arrow) in mice infected by *C. difficile* (CDI group). Scale bars, 100 µm. **(D)** Histopathologic score (median, 0-no damage, and 9-intense damage) based on epithelial damage, submucosal edema, and infiltration of inflammatory cells. Two-tailed non-parametric Mann–Whitney U-test. **(E)** MPO levels (mean ± s.e.m.) in cecum content, cecum, and colon samples from CDI and CDI+pentamidine mice at day 3 p.i. measured by ELISA. Unpaired two-tailed *Student’s t* test.

Since S100B levels are increased during CDI in mice and humans, we next sought the influence of this mediator in *C. difficile* shedding, diarrhea, and intestinal damage. We used pentamidine, a known S100B inhibitor (McKnight et al., 2012; Costa et al., 2019), as a pharmacologic blocker to inhibit S100B activity. Pentamidine at 40 mg/kg was selected based on a dose-ranging study (**Additional File: Figure S3**). As shown in **Figure 2A**, S100B inhibition did not affect *C. difficile* shedding in stools from infected mice. As we have previously shown (Costa et al., 2019), pentamidine treatment does not prevent the initial weight loss from acute infection (**Additional File: Figure S4A**). However, S100B inhibition decreased diarrhea severity on days 2 (p = 0.01) and 3 (p = 0.04) p.i. (**Figure 2B** and **Additional File: Figure S4B**), as well as on days 4 (p = 0.007) and 6 (p = 0.01) p.i. (**Additional File: Figure S4B**). Of note, all non-treated infected mice developed severe diarrhea whereas most of S100B inhibitor-treated infected mice exhibited only mild to moderate diarrhea (**Figure 2B**).

By histopathology, CDI resulted in an extensive damage of the epithelial layer, edema, and intense inflammatory cell infiltration in the cecum and colon (**Figure 2C**), resulting in higher histopathologic scores (median of score = 9, **Figure 2D**). S100B inhibition decreased intestinal tissue damage in both cecum and colon during CDI, resulting in reduction of histopathologic scores (median of score = 1, **Figures 2C, D**).

In addition, we found that inhibition of S100B decreased the MPO levels in cecum content (p = 0.001), cecum (p = 0.001), and colon (p = 0.01) tissues during CDI compared to non-treated infected mice (**Figure 2E**), indicating a reduction in neutrophil recruitment during CDI.

Taken together, these findings indicate that the S100B inhibition-mediated protective effect in preventing diarrhea and intestinal damage occurs *via* controlling host inflammatory response and not by decreasing pathogen burden.

### S100B Inhibition Abrogates the Increased Synthesis of Pro-Inflammatory Mediators and Promotes the Expression of SOCS2 and the Anti-Apoptotic Gene Bcl-2 in *C. difficile-*Infected Mice

**Figure 3:**
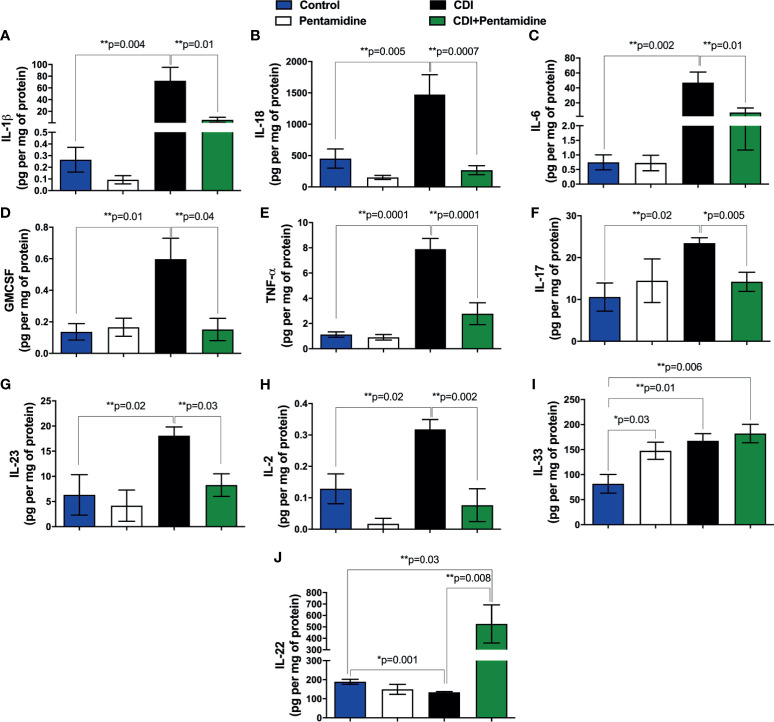
S100B modulates the release of pro-inflammatory mediators and tissue repair cytokines during CDI in mice. Levels of **(A)** IL-1β, **(B)** IL-18, **(C)** IL-6, **(D)** GMCSF, **(E)** TNF-α, **(F)** IL-17, **(G)** IL-23, **(H)** IL-2, **(I)** IL-33, and **(J)** IL-22 in colonic tissues from uninfected (control), uninfected receiving pentamidine (40 mg/kg, pentamidine), non-pretreated *C. difficile*-infected (CDI), and pentamidine-pretreated *C. difficile-*infected (CDI+pentamidine) mice at day 3 p.i. were measured by ELISA. Data are mean ± s.e.m. **ANOVA followed by Turkey test was used. *Unpaired two-tailed Student’s t test.

S100B is an important glial factor that exhibits a dual effect in the gut, stimulating the survival or death of enteric neurons and functioning as a proinflammatory cytokine during the inflammatory response. We investigated whether S100B regulates the synthesis of IL-1β, IL-18, IL-6, GMCSF, TNF-α, IL-17, IL-23, IL-2, and IL-22, all mediators involved in CDI pathogenesis, by pharmacologic blockage using pentamidine. We found that S100B inhibition decreased the colonic levels of IL-1β, IL-18, IL-6, GMCSF, TNF-α, IL-17, IL-23, and IL-2, but not IL-33, during CDI (p < 0.05, **Figures 3A–I**). On the other hand, S100B inhibition increased the colonic levels of IL-22 during infection (p = 0.008, **Figure 3J**).

**Figure 4:**
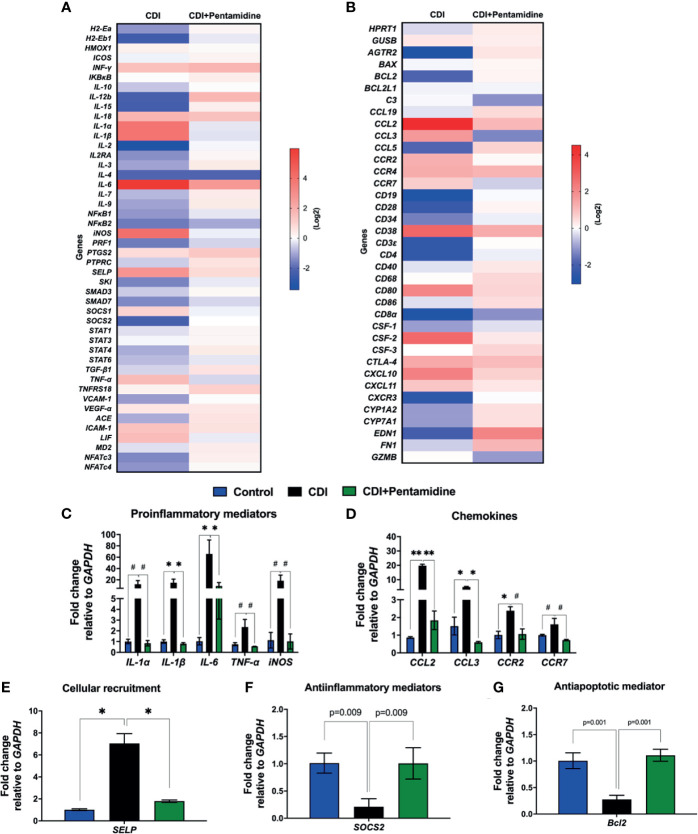
S100B inhibition upregulates anti-inflammatory (*SOCS2*) and antiapoptotic mediators (*Bcl2*) and downregulates inflammatory mediators during CDI. **(A, B)** Heat map of the cDNA microarray analysis of colonic tissues from non-pretreated *C. difficile*-infected (CDI) and pentamidine-pretreated *C. difficile-*infected (CDI+pentamidine) mice at day 3 p.i. Expression of the genes is normalized to median of the control, log 2 scale. TaqMan qPCR analysis of **(C)** proinflammatory **(D)** chemokine, **(E)** cellular recruitment (*SELP*), **(F)** anti-inflammatory *SOCS2*, and **(G)** antiapoptotic *Bcl2* mediators. Data are mean ± s.e.m. **(C–E)** ^#^p < 0.05, *p < 0.01, **p < 0.001. ANOVA followed by Turkey test was used.

Using a TaqMan qPCR, we found that blockage of S100B activity downregulated the expression of proinflammatory mediators (*IL-1α*, *IL-1β*, *IL-6*, *TNF-α*, and *iNOS*), chemokines (*CCL2* and *CCL3*), chemokine receptors (*CCR2* and *CCR7*), and cellular recruitment-related molecule (*SELP*, selectin P) transcripts and upregulated the anti-inflammatory (*SOCS2*, suppressor of cytokine signaling 2) and antiapoptotic (*Bcl-2*, B-cell lymphoma 2) mediators in infected mice (p < 0.05, **Figures 4A–G**).

Taken together, these findings suggest that S100B has a modulatory role in the intestinal inflammatory response during CDI.

### *C. difficile* Toxins, TcdA and TcdB, Upregulate S100B and IL-6 in Rat Enteroglial Cell Lineage (EGC/PK060399) in a Time-Dependent Manner

Because S100B is predominantly secreted by EGCs during normal conditions (Rao et al., 2015), we investigated the effect of *C. difficile* toxins, TcdA and TcdB, in a rat EGC line (EGC/PK060399), which has been largely used (Bauman et al., 2017; Fettucciari et al., 2017; Macchioni et al., 2017; Chen et al., 2018; Fettucciari et al., 2018; Kimono et al., 2019). We tested varying concentrations of TcdA and TcdB and analyzed cell viability and morphology. We selected TcdA 50 ng/ml and TcdB 1 ng/ml as the optimal concentrations for the succeeding *in vitro* studies (**Additional File: Figure S5**).

**Figure 5:**
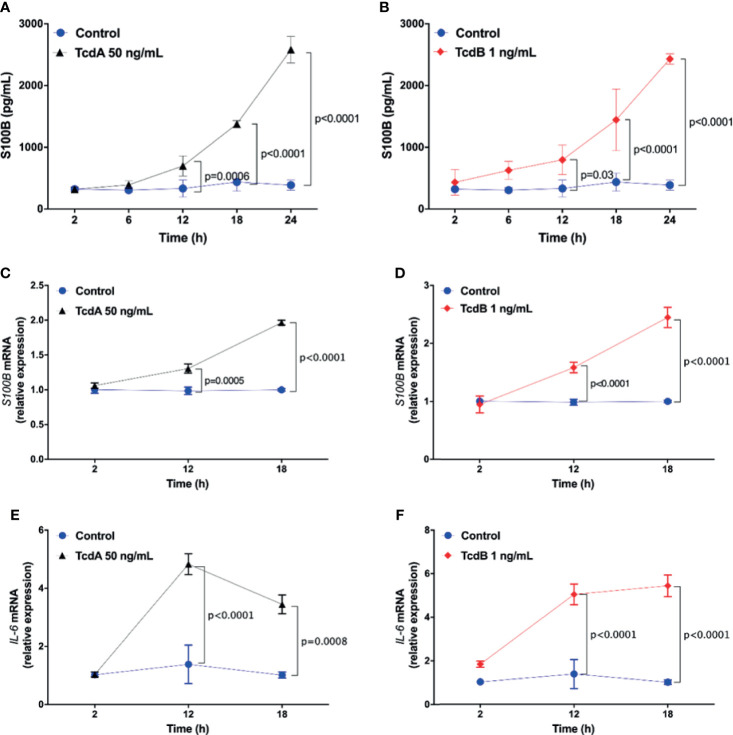
TcdA and TcdB increase S100B-dependent upregulation of *IL-6* expression in enteroglial cells (EGC/PK060399). **(A, B)** Levels of S100B (mean ± s.e.m) released by ELISA, **(C, D)** *S100B*, and **(E, F)** *IL-6* gene expression (mean ± s.e.m) by qPCR in enteroglial cells (EGC/PK060399) challenged with **(A, C, E)** TcdA and **(B, D, F)** TcdB (n = 4) with TcdA and TcdB (n = 4). **(A–F)** Cells receiving only supplemented DMEM were applied as a control. **(A–F)** ANOVA followed by Sidak’s multiple-comparison test was used.

Given that we found increased S100B in *C. difficile-*infected human and mice, we investigated whether TcdA and TcdB stimulate the expression and secretion of S100B in the EGC lineage (EGC/PK060399). We found that TcdA and TcdB increased S100B release by EGC (EGC/PK060399) in a time-dependent manner (p < 0.0001, **Figures 5A, B**). In addition, we found that TcdA and TcdB upregulated *S100B* mRNA in EGCs after 12 h of incubation (p < 0.0001, **Figures 5C, D**).

Since most inflammatory mediators have been shown to be increased by *C. difficile* toxins in several types of cells, such as epithelial cells, neutrophils, macrophages, and T cells (Ng et al., 2010; Cowardin et al., 2015), we investigated whether TcdA and TcdB induce *IL-6* transcript expression in EGC (EGC/PK060399). TcdA and TcdB upregulated the expression of *IL-6*, a severity marker of CDI in humans and mice (Yu et al., 2017), at 12 h of incubation (p < 0.0001, **Figures 5E, F**).

These findings indicate that *C. difficile* toxins upregulate the expressions of both IL-6 and S100B in EGC (EGC/PK060399).

### RAGE Activation Is Involved in TcdA- and TcdB-Induced S100B and IL-6 Upregulation

**Figure 6:**
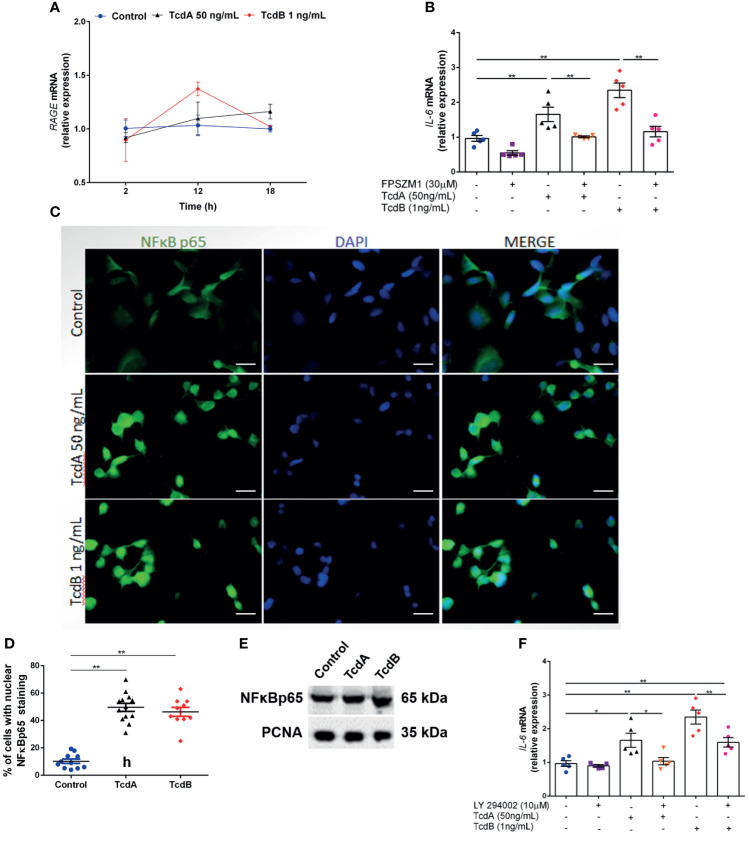
Blockade of the S100B receptor and PI3K decreases *C. difficile* toxin-induced IL-6 upregulation in EGCs. **(A)** *RAGE* gene expression (mean ± s.e.m) by qPCR in enteroglial cells (EGC/PK060399) challenged with TcdA and TcdB (n = 4). **(B)** Analysis of *IL-6* gene expression (mean ± s.e.m) by qPCR in enteroglial cells (EGC/PK060399) challenged with TcdA and TcdB for 18 h in the presence or absence of 30 μM FPSZM1 (a RAGE antagonist) which was added 1 h prior to *C. difficile* toxin challenge. **(C)** Representative photomicrographs of NFκBp65 (green) immunostaining and DAPI (blue) nuclear staining in enteroglial cells (EGC/PK060399) exposed to TcdA and TcdB after 18 h of incubation. **(D)** Percentages of cells (mean ± s.e.m) with positive nuclear NFκBp65 staining under different experimental conditions at 18 h of incubation with TcdA and TcdB. **(E)** Western blot (WB) bands of NFκB p65 and PCNA in nuclear extract fraction of enteroglial cells (EGC/PK060399) exposed to TcdA and TcdB at 18 h of incubation. **(F)** Analysis of *IL-6* gene expression (mean ± s.e.m) by qPCR in enteroglial cells (EGC/PK060399) challenged with TcdA and TcdB for 18 h in the presence or absence of 10 μM LY294002 (a PI3K inhibitor) which was added 1 h prior to *C. difficile* toxin challenge. **(A–F)** Cells receiving only supplemented DMEM was applied as a control. **(B, F)** Experiments were performed with the same negative (Control) and positive controls (TcdA and TcdB). **p < 0.0001. ANOVA followed by **(B, D, F)** Turkey test was used. *p < 0.01.

To study the signaling pathway involved in S100B-induced *IL-6* expression in EGCs, we asked whether the receptor for advanced glycation end products (RAGE), a known receptor for S100B extracellular function, is activated during intoxication. While EGC/PK060399 expressed RAGE, we found that TcdA and TcdB did not alter *RAGE* expression in this cell line (**Figure 6A**). However, inhibition of RAGE activation using a RAGE antagonist, FPSZM1, reduced the *IL-6* expression induced by TcdA and TcdB (p < 0.0001, **Figure 6B**). These results suggest that the TcdA- and TcdB-induced *IL-6* upregulation in EGC (EGC/PK060399) occurs through RAGE activation.

### TcdA and TcdB Promote NFκB Activation, and PI3K Inhibition Suppresses TcdA- and TcdB-Induced IL-6 Upregulation

In vascular inflammatory conditions, RAGE activation leads to NFκB activation, which in turn regulates the transcription of proinflammatory cytokines, such as IL-6 (Brasier, 2010). We found that TcdA and TcdB increased the nuclear translocation of NFκBp65 in EGC (EGC/PK060399) at 18 h of incubation (p < 0.0001, **Figures 6C–E**). TcdA or TcdB increased nuclear phosphorylated NFκBp65 even at 2 and 4 h in EGCs (**Additional File: Figures S6A, B**). Since RAGE activation is known to initiate PI3K/AKT signaling, which acts upstream of the NFκB pathway, we inhibited PI3K using LY294002, which accentually decreased nuclear phosphorylated NFκBp65 in EGCs challenged with TcdA or TcdB at 4 h of incubation (**Additional File: Figure S7**). LY294002 notably reduced the TcdA- and TcdB-induced *IL-6* upregulation in EGC (EGC/PK060399) (p < 0.0001, **Figure 6F**).

Taken together, these results indicate that the RAGE/PI3K/NFκB pathway is involved in the induction of *IL-6* expression by TcdA and TcdB in EGC (EGC/PK060399).

## Discussion

In our study, we observed an increased expression of S100B in all layers of colonic tissues from humans and mice infected with *C. difficile*, as well as in fecal samples of patients with CDI, suggesting that this factor may be involved in CDI. Others have found that S100B activity is likewise involved in a variety of inflammatory diseases, such as ulcerative colitis (Celikbilek et al., 2014), celiac disease (Esposito et al., 2007), and chemotherapeutic-induced intestinal inflammation (Nogueira et al., 2017; Costa et al., 2019). Although S100B has been shown to be synthetized by other cells, such as CD8+T cells, NK cells, and neurons (Steiner et al., 2011; Miki et al., 2013; Cristovao et al., 2018) during inflammation, EGCs are the main source of S100B during homeostasis (Rao et al., 2015). It has been shown that EGCs are reduced in intestinal mucosa tissue during dysbiosis (Kimono et al., 2019); similarly, we showed that microbiota disruption by antibiotic exposure trended to decrease S100B levels in uninfected mice on day 1 p.i. Inflamed colonic tissues from patients with CDI have been shown to have an elevated expression of GFAP, another enteric glial factor (von Boyen et al., 2011). For the first time, we showed that S100B is also elevated during CDI in humans and in mice, suggesting that reactive gliosis, indeed, occurs in *C. difficile*–associated colitis. During intestinal injury, reactive gliosis is an EGC response that protects the neuronal network from damage evoked by the inflammatory response (Burda and Sofroniew, 2014). However, uncontrolled EGC responses to virulence factors from bacteria and proinflammatory mediators released by immune, and even neuronal and glial cells themselves, can lead to tissue damage, including the enteric nervous system.

CDI in humans and mice is represented by severe diarrhea that is associated with body weight loss. In mice, the S100B blocker pentamidine effectively reduced the severity of diarrhea but did not improve the body weight loss induced by CDI on days 2–7 p.i. (**Additional File: Figure S4A**). It is known that pentamidine decreases the appetite when administered in major doses (Costa et al., 2019), which could explain its reduced effect on weight loss. However, non-severe diarrhea was detected in mice with CDI receiving pentamidine (**Additional File: Figure S4B**). Pentamidine has been broadly used to block S100B in mouse models (Esposito et al., 2012; Cirillo et al., 2015; Di Sante et al., 2020).

CDI is also characterized by an intense intestinal tissue inflammatory reaction with the involvement of proinflammatory cytokines produced by epithelial cells and immune cells (El Feghaly et al., 2013; Walker et al., 2013). Our study showed that blocking S100B, the syntheses of IL-1β, IL-18, IL-6, GMCSF, TNF-α, IL-17, IL-23, and IL-2 are markedly decreased, and neutrophil recruitment is inhibited as shown by MPO measurement in colonic tissues from mice with CDI. These inflammatory mediators have previously been implicated in CDI pathogenesis. IL-1β has been shown to play a dual role during CDI. Its protective effect against infections occurs by upregulating chemotactic chemokines, recruiting neutrophils to inflammatory sites, and contributing to bacterial clearance *in vivo* (Hasegawa et al., 2012; Biondo et al., 2014; Liu Y. H. et al., 2019), and its deleterious effect is by upregulating proinflammatory cytokines such as IL-23 (Cowardin et al., 2015), which is associated with mortality and worse clinical manifestations during CDI in mice (Buonomo et al., 2013). High levels of IL-2, TNF-α, and IL-6 have been previously associated with a poor prognosis in CDI patients (Yu et al., 2017; Abhyankar et al., 2020). IL-17A has been shown to be a protective mediator during CDI; its reduction induced by pretreatment with the S100B inhibitor may be a result of the downregulation of pro-inflammatory mediators such as IL-1β and IL-23, both of which are known as inductors of this cytokine (Shibata et al., 2007; Sutton et al., 2009). On the other hand, blockage of S100B resulted in the downregulation of pro-inflammatory response and upregulation of *SOCS2*, an anti-inflammatory mediator, and *Bcl2*, an antiapoptotic mediator, as well as IL-22 protein. SOCS2 is known as a modulator of the immune system by decreasing NFκB activation (Monti-Rocha et al., 2018). Our findings suggest that S100B induces NFκB activity during CDI, likely upregulating genes promoted by this transcription factor: *IL-1α*, *IL-1β*, *IL-6*, *TNF-α*, *iNOS*, *CCL2*, *CCL3*, *CCR2*, and *CCR7*. IL-22, which is secreted mainly by innate lymphoid cells type 3 (ILCs3) (Fachi et al., 2020), has been shown to play an important protective role in promoting epithelial regeneration and regulating intestinal microbiota during CDI (Fachi et al., 2020; Nagao-Kitamoto et al., 2020). Although IL-22 is regulated by IL-1β (Seshadri et al., 2018), other mediators such as TGFβ, which tended towards an increase in CDI mice treated with pentamidine (**Figure 3A**) and was shown to be protective against epithelial damage induced by *C. difficile* toxins (Tinoco-Veras et al., 2017), have also been involved in regulating IL-22 levels (Johnson et al., 2013; Perez et al., 2020). In our study, S100B appears to be indirectly involved with IL-22 activity, and thus, it is unclear whether the beneficial effect of blocking S100B activity is related to IL-22 expression during CDI.

S100B is a ligand to TLR4 and RAGE, which are present in a diversity of cell types. RAGE has a ubiquitous expression in the gut and has been identified in enteric neurons (Costa et al., 2019), epithelial cells (Body-Malapel et al., 2019), macrophage (Liu T. et al., 2019), neutrophils (Sionov et al., 2019), and T cells (Chen Y. et al., 2008). To investigate the direct effects of *C. difficile* toxins on S100B synthesis by EGCs, we used a rat EGC lineage (EGC/PK060399) which has been shown to exhibit similar morphology and functional properties to primary enteric glial cells (Ruhl et al., 2001) and is largely used (Bauman et al., 2017; Fettucciari et al., 2017; Macchioni et al., 2017; Chen et al., 2018; Fettucciari et al., 2018; Kimono et al., 2019). Of note, EGCs from mouse, rat, and human are known to have similar functional and morphologic properties (Soret et al., 2013). We found that both TcdA and TcdB upregulated S100B expression and increased its secretion in the EGC lineage (EGC/PK060399), which was associated with elevated *IL-6* mRNA expression. IL-6 is a pleiotropic cytokine, a predictor of CDI severity and mortality (Abhyankar et al., 2020), which has dual effects, promoting cell survival and proinflammatory responses (Brasier, 2010; Codeluppi et al., 2014). Other inflammatory mediators, such as nitric oxide, had been shown to be stimulated by S100B in EGCs (Cirillo et al., 2011).

Although neither TcdA nor TcdB altered the expression of RAGE, inhibition of this receptor by FPSZM1 (Bongarzone et al., 2017) attenuated the expression of *IL-6* induced by both toxins in the EGC lineage (EGC/PK060399), suggesting that the S100B–RAGE interaction is involved in regulating the expression of IL-6. In line with our results, a previous study reported that RAGE activation in fibroblasts results in the upregulation of proinflammatory cytokines, such as IL-6, *via* NFκB (McKnight et al., 2012). Here, we also demonstrated that activation of PI3K/NFκB is necessary for *IL-6* expression in the presence of either TcdA or TcdB. Regulation of *IL-6* by activation of PI3K/NFκB has been reported in cancer cells, as well as astrocytes (Xie et al., 2004; Brasier, 2010). Therefore, it appears that S100B/RAGE/PI3K/NFκB is the signaling pathway involved in TcdA- and TcdB-induced upregulation of *IL-6* in EGCs.

Although most of the effects of both toxins on eukaryotic cells are related to the ability of these toxins to inhibit Rho GTPases, such as Rac1 and Cdc42 (Yuan et al., 2015; Tao et al., 2016), our findings suggest that other pathways such as S100B/RAGE/PI3K/NFKB are also involved in these effects. S100B/RAGE had been shown to change astrocyte morphology and microglial migration which was Rac1/Cdc42 dependent (Bianchi et al., 2011; Villarreal et al., 2014). However, more studies are needed to better understand the involvement of Rho GPTase activity to S100B/RAGE signaling in cells challenged with *C. difficile* toxins.

**Figure 7:**
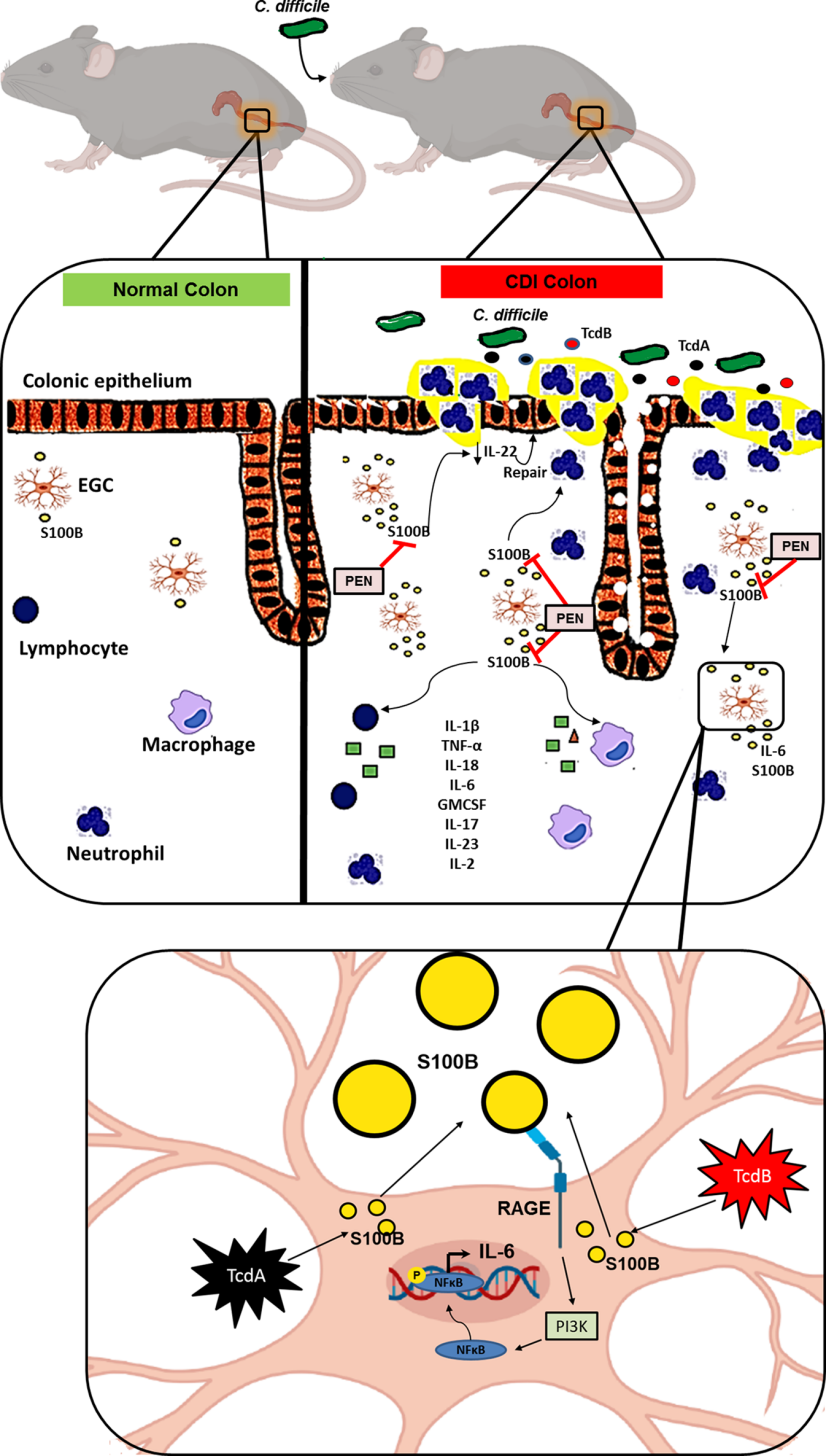
Schematic diagram of the hypothetical role of S100B during *C. difficile* infection. *C. difficile* releases TcdA and TcdB which in turn promote epithelial damage and release of S100B by colonic EGCs. S100B stimulates the synthesis and secretion of inflammatory mediators (IL-1β, TNF-α, IL-18, IL-6, GMCSF, IL-17, IL-23, IL-2) promoting recruitment of immune cells, such as neutrophils, macrophages, and T cells, resulting in amplification of the *C. difficile* toxin-induced colonic damage. TcdA and TcdB induce *IL-6* expression *via* S100B/RAGE/PI3K/NFκB. S100B also impairs epithelial integrity during CDI by decreasing IL-22 production, thereby hampering repair of the epithelium. Inhibition of S100B by pentamidine (PEN) blocks these events mediated by S100B during CDI.

Our study is the first to show that S100B, which is increased during CDI in humans and mice, is an important regulator of inflammatory response during CDI, thereby functioning as a key mediator of the intestinal tissue injury and diarrhea by upregulating a variety of proinflammatory mediators (IL-1β, IL-18, IL-6, GMCSF, TNF-α, IL-17, IL-23, and IL-2) and downregulating protective mediators (SOCS2, IL-22, and Bcl-2). In addition, we demonstrated that *C. difficile* toxins (TcdA and TcdB) upregulated *S100B* and *IL-6* in the EGC lineage (EGC/PK060399) and S100B is a mediator to the induction of *IL-6* gene expression by activating RAGE/PI3K/NFκB (**Figure 7**). While our study focused on the role of S100B activity during active infection, further studies on how manipulation of the intestinal S100B signaling could ameliorate CDI outcomes (such as recurrence and intestinal dysfunction) are needed.

## Data Availability Statement

The datasets presented in this study can be found in online repositories. The names of the repository/repositories and accession number(s) can be found in the article/**Supplementary Material**.

## Ethics Statement

Fecal samples from patients with diarrhea caused by CDI and non-CDI were obtained from the clinical microbiology laboratory of University of Virginia after Institutional Review Board (IRB) protocol approval (Protocol number: 20813). The patients/participants provided their written informed consent to participate in this study. The protocol has been approved by the committee on the Ethics of Animal Experiments of the University of Virginia (Protocol number: 4096).

## Author Contributions

DC participated in design and performed experiments, analyzed the data, and wrote the manuscript. DB, RG, JF, JS, PM, SL, GK, CM, and VV helped in acquisition of data and review of manuscript. VM-N participated in initial experimental design and helped to revise the manuscript. CW, the principal investigator in the laboratory where the *in vitro* experiments, mouse infection, and human tissue studies were performed, participated in experimental design, supervised the project, and reviewed the manuscript. GB conceptualized the main ideas, supervised the study, and reviewed the manuscript. All authors contributed to the article and approved the submitted version.

## Funding

This study was funded by CAPES/Procad grant number 23038.014449/2016-07, PRONEX CNPq/FUNCAP grant number PR2-0101-00060.01.00/15, CAPES/PDSE 88881.134019/2016-01, and CAPES/PNDP (001) 88887.358214/2019-00. This study was also supported by the University of Virginia Infectious Disease Seed Grant 148142.CA6T.SS00178.40775.

## Conflict of Interest

The authors declare that the research was conducted in the absence of any commercial or financial relationships that could be construed as a potential conflict of interest.

## Publisher’s Note

All claims expressed in this article are solely those of the authors and do not necessarily represent those of their affiliated organizations, or those of the publisher, the editors and the reviewers. Any product that may be evaluated in this article, or claim that may be made by its manufacturer, is not guaranteed or endorsed by the publisher.
