# Supplementary material for: S100B Inhibition Attenuates Intestinal Damage and Diarrhea Severity During Clostridioides difficile Infection by Modulating Inflammatory Response
Source: Front Cell Infect Microbiol. 2021 Sep 10;11:739874. doi: 10.3389/fcimb.2021.739874 (PMC8461106; doi:10.3389/fcimb.2021.739874)
Supplement: Supplementary file 1 [file DataSheet_1.pdf]

S100B Inhibition Attenuates Intestinal Damage and Diarrhea Severity During *Clostridioides difficile* Infection by Modulating Inflammatory Response

Deiziane V.S. Costa[1,2,3], Vivaldo Moura-Neto[4], David T. Bolick[2], Richard L. Guerrant[2], Jibraan A. Fawad[2], Jae H. Shin[2], Pedro H.Q.S. Medeiros[5], Solanka E. Ledwaba[6], Glynis L. Kolling[2], Conceição S. Martins[1], Venkat Venkataraman[7], Cirle A. Warren[2&*], and Gerly A.C. Brito[1,3*&]

& Cirle A. Warren and Gerly A.C Brito provided equal contribution

[1]Department of Physiology and Pharmacology, Faculty of Medicine, Federal University of Ceará, Fortaleza, Ceará, Brazil; [2]Division of Infectious Diseases and International Health, University of Virginia, Charlottesville, Virginia, USA; [3]Department of Morphology, Faculty of Medicine, Federal University of Ceará, Fortaleza, Ceará, Brazil; [4]Paulo Niemeyer Brain Institute, Federal University of Rio de Janeiro, UFRJ, Rio de Janeiro, Rio de Janeiro, Brazil; [5]Department of Microbiology, Faculty of Medicine, Federal University of Ceará, Fortaleza, Ceará, Brazil; [6]University of Venda, Thohoyandou, Limpopo Province, South Africa; [7]Department of Cell Biology and Neuroscience, Department of Rehabilitation Medicine, Rowan University School of Osteopathic Medicine, Stratford, New Jersey, USA.

*** CONTACT:** Gerly A. C. Brito, gerlybrito@hotmail.com, Federal University of Ceara, Fortaleza, CE, Brazil; Cirle A. Warren, ca6s@virginia.edu, University of Virginia, Charlottesville, VA, USA.

Figure S1

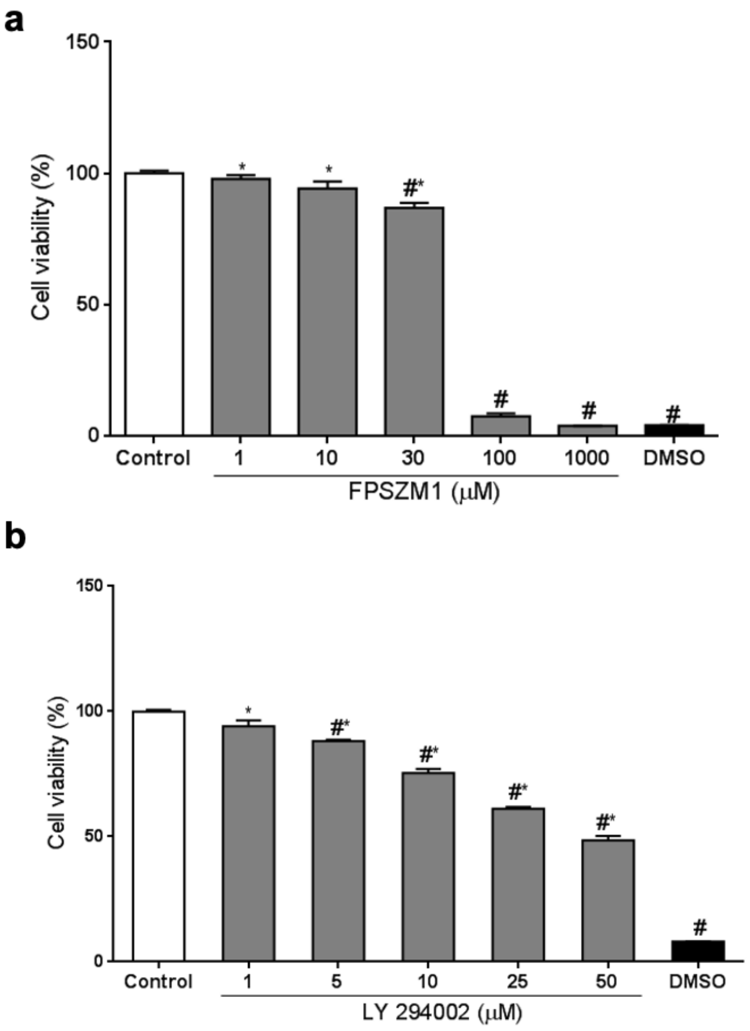

Effects of FPSZM1 and LY294002 on enteroglial cell (EGC/PK060399) viability. Enteroglial cell (EGC/PK060399) viability were assessed by MTT assay after 18h incubation with (a) FPSZM1 (1, 10, 30, 100 and 1000 μM), a RAGE antagonist, and (b) LY294002 (1, 5, 10, 25 and 50 μM), a PI3K inhibitor. DMSO was used as a death control group. Data are presented as the mean ± s.e.m (n = 6). *p<0.0001 versus control group and # p<0.0001 versus DMSO group. One-way ANOVA followed by Turkey test.

Figure S2

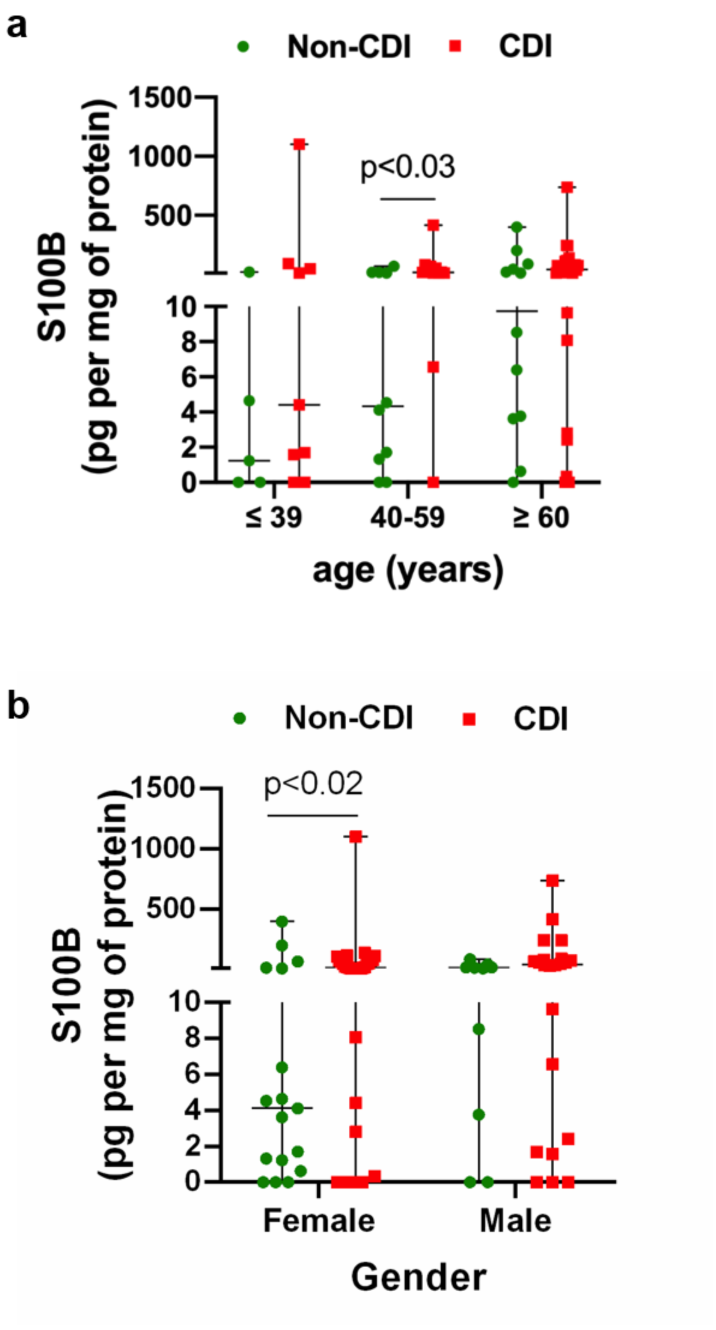

Differences in faecal S100B levels between patients with diarrhea caused by CDI and non-CDI is age and gender dependent. (a-b) S100B levels in faecal samples were measured by ELISA. (a) S100B levels in patients with diarrhea caused by CDI and non-CDI according to age (≤39, 40-59 and ≥60 years old). (b) S100B levels in patients with diarrhea caused by CDI and non-CDI according to gender. Data are median ± s.d. Two-tailed non-parametric Mann-Whitney U-test.

Figure S3

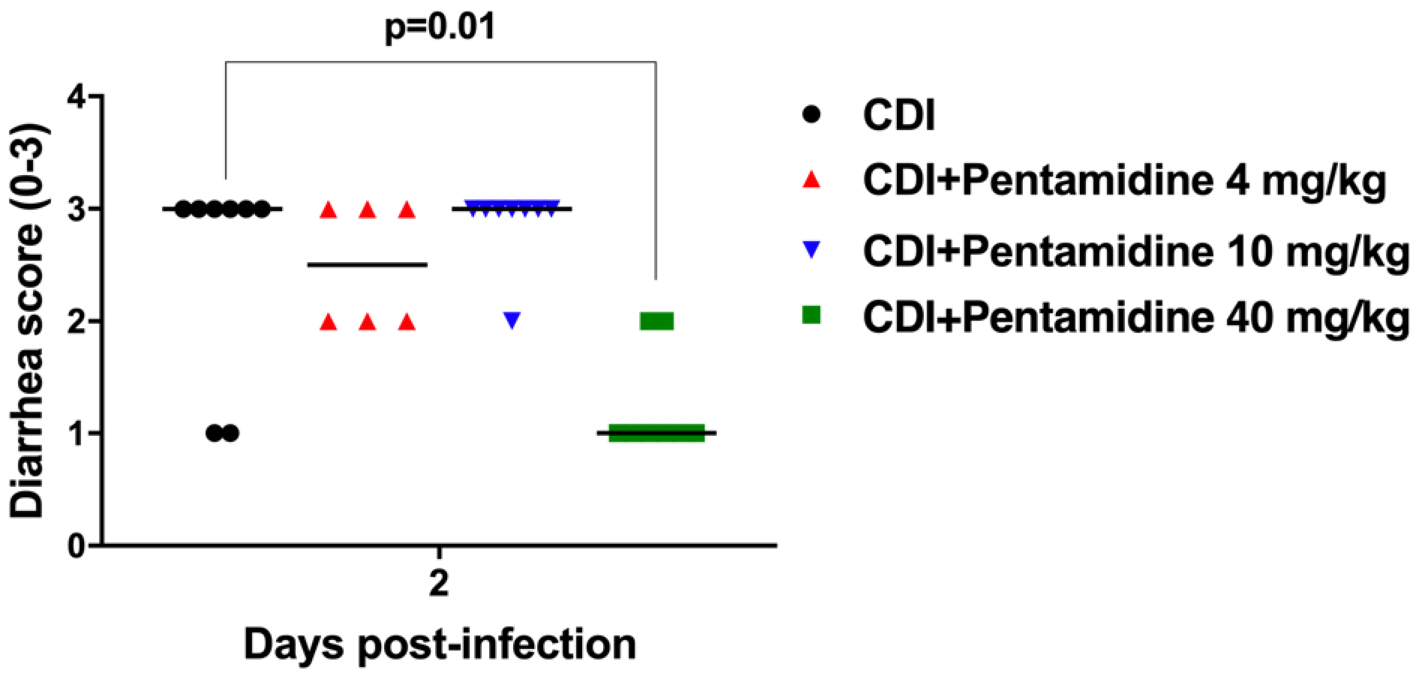

Effect of different doses of pentamidine, a S100B inhibitor, **on prevention of CDI-induced diarrhea in mice.** Mice were infected with 10^5 vegetative *C. difficile* (VPI10463 strain) and treated with pentamidine (4, 10 and 40 mg/kg, i.p., once-daily dose for three days, CDI+pentamidine group) or non-treated (CDI group). Diarrhea score (median) of CDI and CDI+pentamidine mice. Two-tailed non-parametric Mann-Whitney U-test.

Figure S4

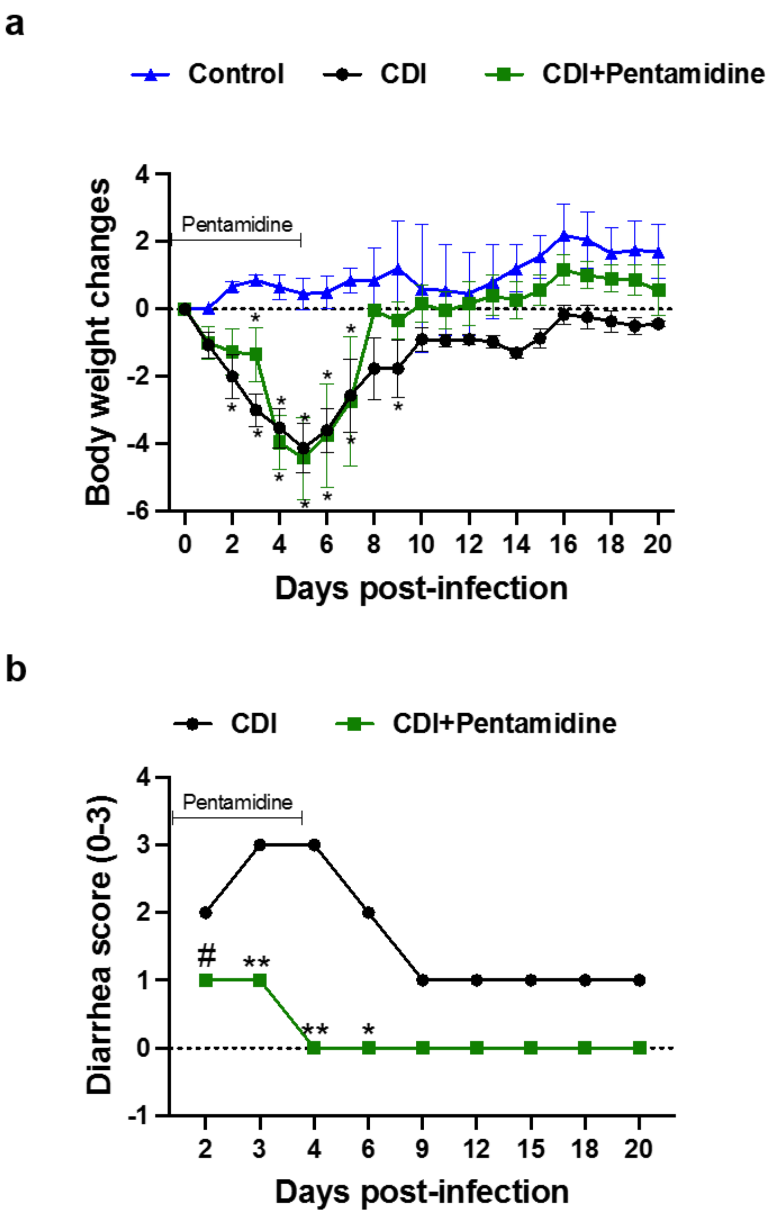

(a) Change in body weight of C57BL/6 mice non treated infected with C. difficile (CDI), uninfected (Control) and pentamidine treated infected (CDI+pentamidine) (n=6/group). Line graphs represents mean±SEM. *p<0.03 versus control group. (b) Diarrhea score (median) of CDI and CDI+Pentamidine mice. Non-parametric Mann-Whitney U-test. #p= 0.04, *p=0.01, **p=0.007.

Figure S5

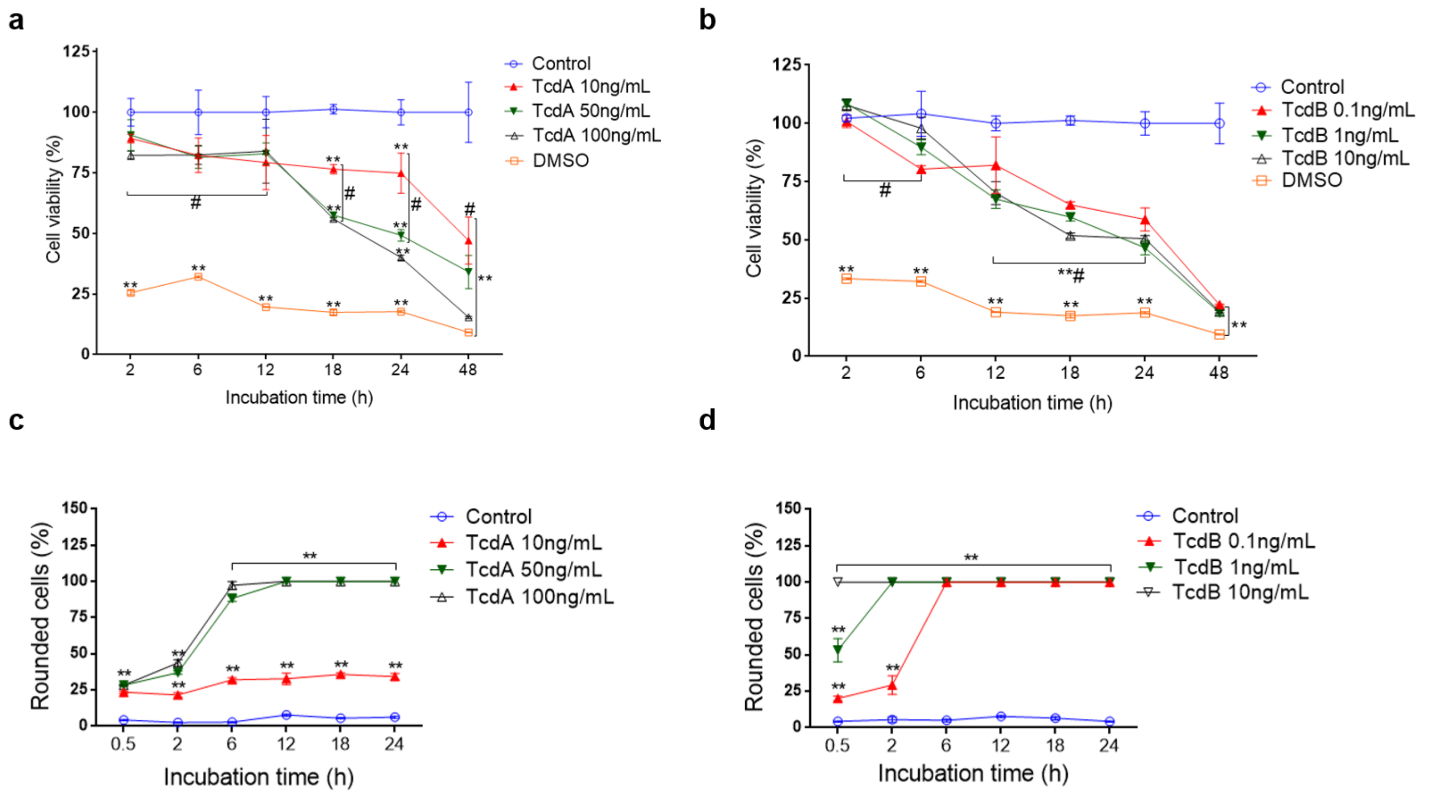

TcdA and TcdB induce enteroglial cell (EGC/PK060399) death and morphology alteration in a time-dependent manner. (a,b) Effect of (a) TcdA (10, 50 and 100 ng/mL) and (b) TcdB (0.1, 1 and 10 ng/mL) on cell viability in enteroglial cell (EGC/PK060399). Cell viability was determined by the MTT assay. Data are presented as the mean ± s.e.m. (n = 6). **$p<0.0001$ versus control group and # $p<0.0001$ versus DMSO group. (c, d) Morphology analysis showing the percentage mean ± SEM of rounded cells. 100 cells were counted for each experimental condition (n=6). **$p<0.0001$ versus control group (Cells receiving only supplemented DMEM). ANOVA followed by Sidak's multiple comparisons test was used.

Figure S6

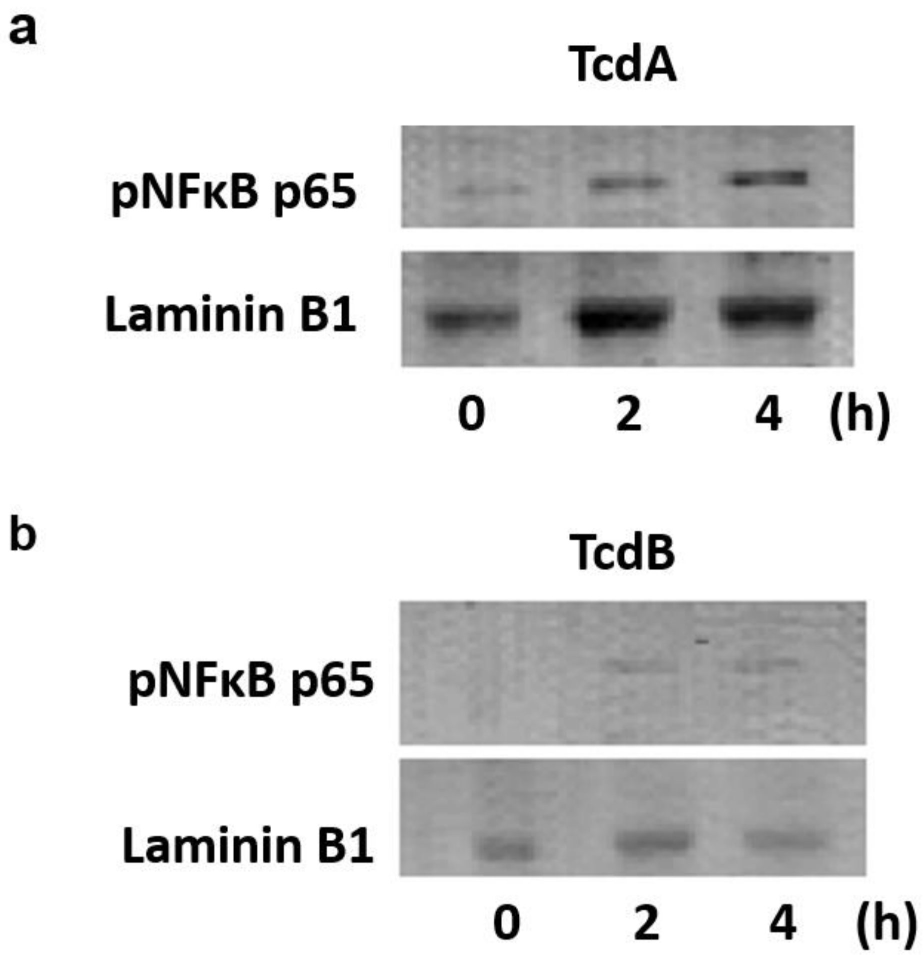

(a, b) Western blot (WB) bands of phosphorylated NFκB p65 and laminin B1 in nuclear extract fraction of enteroglial cell (EGC/PK060399) exposed to TcdA (a) and TcdB (b) at 0, 2 and h of incubation.

Figure S7

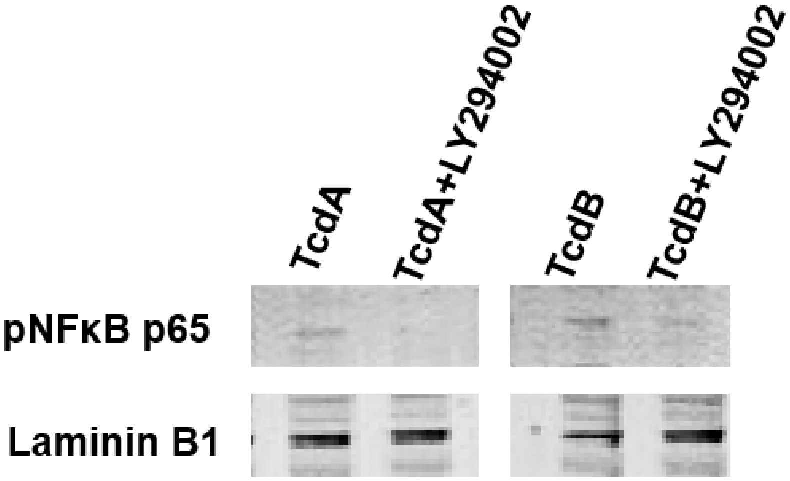

Western blot (WB) bands of phosphorylated NFκB p65 and laminin B1 in nuclear extract fraction of enteroglial cell (EGC/PK060399) exposed to TcdA and TcdB at 4 h of incubation in the presence or absence of LY294002 (a PI3K inhibitor).

Table S1-Primers used for the study

Primer name	Sequence (5'->3')
S100B	Forward: TCCAGGGAGAGAGGGTGACAA<br><br>Reverse: CTTCCTGCTCTTTGATTTCCTCC
IL-6	Forward: GCCAGAGTCATTCAGAGCAATA<br><br>Reverse: GTTGGATGGTCTTGGTCCTTAG
RAGE	Forward: GGAAGGACTGAAGCTTGGAAGG<br><br>Reverse: TCCGATAGCTGGAAGGAGGAGT
GAPDH	Forward: AGACAGCCGCATCTTCTTGT<br><br>Reverse: CTTGCCGTGGGTAGAGTCAT
